# Noninvasive Characterization of Functional Pathways in Layer-Specific Microcircuits of the Human Brain Using 7T fMRI

**DOI:** 10.3390/brainsci12101361

**Published:** 2022-10-07

**Authors:** Gopikrishna Deshpande, Yun Wang

**Affiliations:** 1 AU MRI Research Center, Department of Electrical & Computer Engineering, Auburn University, Auburn, AL 36849, USA; 2 Department of Psychological Sciences, Auburn University, Auburn, AL 36849, USA; 3 Alabama Advanced Imaging Consortium, University of Alabama, Birmingham, AL 35294, USA; 4 Center for Neuroscience, Auburn University, Auburn, AL 36849, USA; 5 Key Laboratory for Learning and Cognition, School of Psychology, Capital Normal University, Beijing 100048, China; 6 Department of Psychiatry, National Institute of Mental Health and Neurosciences, Bangalore 560029, India; 7 Centre for Brain Research, Indian Institute of Science, Bangalore 560012, India; 8 Department of Heritage Science and Technology, Indian Institute of Technology, Hyderabad 502285, India; 9 Department of Psychiatry, Columbia University, New York, NY 10027, USA; 10 Department of Psychiatry and Behavioral Sciences, Duke University, Durham, NC 27708, USA; 11 Department of Electrical and Computer Engineering, Duke University, Durham, NC 27708, USA

**Keywords:** layer-specific fMRI, high-resolution 7T fMRI, cortical layers, magnocellular lateral geniculate neurons, dynamic directional connectivity, primary visual cortex, corticogeniculate feedback, center-surround inhibition

## Abstract

Layer-specific cortical microcircuits have been explored through invasive animal studies, yet it is not possible to reliably characterize them functionally and noninvasively in the human brain. However, recent advances in ultra-high-field functional magnetic resonance imaging (fMRI) have made it feasible to reasonably resolve layer-specific fMRI signals with sub-millimeter resolution. Here, we propose an experimental and analytical framework that enables the noninvasive functional characterization of layer-specific cortical microcircuits. Specifically, we illustrate this framework by characterizing layer-specific functional pathways in the corticogeniculate network of the human visual system by obtaining sub-millimeter fMRI at 7T using a task which engages the magnocellular pathway between the lateral geniculate nucleus (LGN) and the primary visual cortex. Our results demonstrate that: (i) center-surround inhibition in magnocellular neurons within LGN is detectable using localized fMRI responses; (ii) feedforward (LGN → layers VI/IV, layer IV → layer VI) and feedback (layer VI → LGN) functional pathways, known to exist from invasive animal studies, can be inferred using dynamic directional connectivity models of fMRI and could potentially explain the mechanism underlying center-surround inhibition as well as gain control by layer VI in the human visual system. Our framework is domain-neutral and could potentially be employed to investigate the layer-specific cortical microcircuits in other systems related to cognition, memory and language.

## 1. Introduction

The lateral geniculate nucleus (LGN), a small subcortical structure that is part of the thalamus, is a very important relay center in the visual system. There is a dense network of feedforward and feedback projections between the LGN of the thalamus and the primary visual cortex. In the feedforward pathway, three distinct classes of neurons in LGN—magnocellular, parvocellular and koniocellular neurons—receive signals from the retina and transmit them (in parallel) to layer IV and layer VI of the primary visual cortex. In the feedback pathway, the corticogeniculate neurons in layer VI of the primary visual cortex exert influence on those three classes of neurons within the LGN in parallel [1,2,3,4,5,6,7]. These feedforward and feedback parallel processing streams are recognized ubiquitously in the primate visual system, and they are most prominent in the LGN, where three types of neurons (magnocellular, parvocellular and koniocellular neurons) are segregated into distinct strata. Previous studies about the physiology of magnocellular and parvocellular LGN have shown that they have different spatial, temporal, luminance and chromatic stimulus preference [8,9,10,11,12]. The magnocellular neurons respond well for monochromatic, low spatial frequency, high temporal frequency and high contrast visual stimuli with motion compared with parvocellular neurons, which have a preference for color, high spatial frequency, low temporal frequency and low contrast. Consequently, the magnocellular pathway is useful for the perception of “where” information and is processed in the dorsal visual stream, while the parvocellular pathway is useful for the perception of “what” information and is processed in the ventral visual stream. (It is to be noted here that the segregation of magnocellular and parvocellular pathways and their preference for processing specific types of visual information is not mutually exclusive, and a significant amount of cross-talk exists. See Merigan et al [13] for details. However, we only assume that the magnocellular pathway is recruited more than the parvocellular pathway for monochromatic, low spatial frequency, high temporal frequency and high contrast visual stimuli with motion. We do not intend to imply that the magnocellular pathway does not process other types of visual information.) Our focus in this report is on the parts of the magnocellular pathway between LGN and the primary visual cortex.

The corticogeniculate neurons in layer VI of the primary visual cortex are sensitive to visual stimulus orientation and direction of motion. Therefore, the dynamic influence of the feedback to the magnocellular neurons in LGN will depend on how the visual input drives the receptive fields of neurons in layer VI of the primary visual cortex [14]. Although magnocellular neurons in LGN are not selective for orientation or direction, they have circular concentric center-surround receptive fields in which responses from the stimulation of the central receptive field are antagonized by the simultaneous stimulation of the surround receptive field [15,16,17,18]. In particular, the feedback from corticogeniculate neurons in layer VI of the primary visual cortex modulates the strength of this center-surround interaction for moving stimuli. Feedback can make this surround antagonism for moving stimuli stronger and reduce the response further [19]. The magnocellular neurons in LGN seem to be inhibited by projections from neurons in the primary visual cortex via inhibitory neurons in the thalamus, while neurons that project from the LGN to the primary visual cortex are excitatory; in other words, this feedback loop is negative. 

To date, most findings about these feedforward and feedback pathways come from anaesthetized animals with invasive methods such as single unit recordings [2,3,20,21,22,23,24]. Although these studies have certainly increased our knowledge of corticogeniculate feedback mechanisms, it is imperative that they be confirmed in conscious humans. Therefore, we propose an experimental paradigm employing noninvasive imaging methods such as high-resolution functional magnetic resonance imaging (fMRI) at ultra-high fields (7T) coupled with an analysis framework which could potentially be generalized to investigate laminar-level microcircuitry in any part of the human brain. Recent noninvasive studies have successfully employed high-resolution fMRI to investigate the top–down feedback effects on laminar fMRI response in the primary visual cortex [25,26]. However, they infer the effects of feedback and feedforward pathways by clever manipulation of the experimental design by masking some of the effects to indirectly infer these mechanisms. For example, Muckli et al. found that bottom–up feedforward sensory information from external stimuli predominantly activate the mid-layers of the visual cortex while contextual feedback from higher-order regions (inferred by masking the external stimulus) predominantly activated superficial layers. This agrees with what we know about laminar anatomy, i.e., feedback from neurons in higher order regions terminate in superficial layers of the visual cortex. Therefore, using clever experimental manipulation, it is possible to indirectly infer laminar connectivity at a functional level using laminar activation profiles. In contrast, we propose a framework wherein these effects can be directly tested using standard fMRI paradigms and the use of advanced brain connectivity models. To the best of our knowledge, no previous fMRI studies have directly investigated laminar-level feedback or feedforward pathways in the human brain. Here, we pick the example of the magnocellular pathway between LGN and the primary visual cortex (layer IV and layer VI) in the human brain—the feedback from layer VI to LGN, and the feedforward from LGN to layers VI and IV of the primary visual cortex as well as from layer IV to layer VI within the primary visual cortex—since the visual system in general and the lower level circuit involving the LGN and the primary visual cortex in particular are largely similar in humans and animals [27,28,29]. This allows us to form hypotheses based on invasive animal literature that can be tested in humans. However, the proposed framework in itself is generally applicable to any other brain system. This is important given the fact that the similarities between animal and human brains begin to diminish beyond sensory systems and hence, once our method is established in the visual system, it will pave the way for a fine-grained mechanistic understanding of the brain’s laminar-level functional connectome in more complex domains such as cognition and language.

Our objective for this study is to investigate the magnocellular pathway between LGN and the primary visual cortex—the feedback path from corticogeniculate neurons in layer VI of the primary visual cortex to magnocellular neurons in LGN, and the feedforward paths from LGN to layer VI and layer IV of the primary visual cortex, as well as from layer IV to layer VI within the primary visual cortex. We hypothesize that these functional pathways, known from invasive animal studies, can be inferred by employing the proposed analysis pipeline on data acquired noninvasively using fMRI in humans engaging in a visual motion task. In order to test this, we had to overcome three challenges. First, it is difficult to localize human magnocellular LGN neurons because of the small size and deep location of this nucleus within the brain. In order to address this, we designed specific visual stimuli to evoke responses in magnocellular LGN neurons maximally. We used the typical building block of the visual stimulus in the field of visual neuroscience—Gabor patches, which can efficiently activate and match the shape of receptive fields in the visual cortex and therefore help detect center-surround inhibition effects [30,31,32,33,34]. The choice of the Gabor patch sizes for center-surround inhibition effects is based on the work of Murphy and Sillito [16]. The Gabor patch in this study was specifically designed with characteristics, e.g., monochromatic, high contrast, low spatial frequency, and high temporal frequency, which maximally activate magnocellular neurons within LGN and the corresponding corticogeniculate neurons in layer VI within the primary visual cortex [3,35,36,37,38,39,40,41,42]. We then performed the functional mapping of the magnocellular LGN sub-region in the human brain using high-resolution anatomical and functional MRI data acquired at ultra-high field (7T). Second, we needed high-resolution imaging and advanced image processing techniques to resolve cortical layers. Recent advances in high-field functional MRI make it feasible to measure the blood oxygen level dependent (BOLD) signals with sub-millimeter resolution [43,44,45,46]. In addition, several models to construct laminar profiles have been proposed, including the equidistant laminae model [47], the equipotential method with Laplacian equation [48] and the equal-volume model [49]. In this study, we adopted the equidistant laminae model, which keeps a relatively fixed distance to the cortical boundaries, because equidistant stratification contains a broad isocontour that follows the stria of Gennari everywhere in the primary visual cortex [47]. The drawback of the equipotential model is that the Laplacian equation does not match the anatomical layers observed from high-resolution MRI. As regards the equal-volume model, it does not provide improvement over the equidistant model because of its weak estimation of local cortical curvature [49]. Third, we had to use a dynamic effective connectivity model to unveil the directional influences between layers in the primary visual cortex and LGN. We used a state-of-the-art dynamic Granger causality [50,51,52,53,54] method to quantify the dynamic feedforward and feedback pathways between magnocellular LGN and primary visual cortex. The method involves building a dynamic multivariate autoregressive model using time series extracted from LGN as well as different layers of the visual cortex. By allowing the model coefficients to vary as a functional of time, we can estimate them using a Kalman filter framework. The variation of these coefficients with time allows us to bin them into different experimental conditions corresponding to different times of the experiment and statistically compare the bins to make inferences about the relative strength of effective connectivity across different experimental conditions. The advantage with this method is that it is completely data driven without a need to hypothesize and compare competing models (as in DCM or structural equation modeling).

## 2. Materials and Methods

### 2.1. Subjects

Twenty adult subjects (10 males, 10 females; 24.5 ± 3.3 years of age) participated in this study. All subjects had normal eyesight or corrected-to-normal visual goggles. All subjects provided informed consent, and the experimental protocols were approved by the Auburn University Institutional Review Board. All subjects were scanned on a 7 Tesla Siemens MAGNETOM MRI scanner. 

### 2.2. Visual Stimuli and Task

In this study, we designed specific visual stimuli—six different sizes of rightward moving sine wave gratings (Gabor patches), which can maximally elicit the BOLD response of magnocellular LGN and corticogeniculate neurons in layer VI of the primary visual cortex. Gabor patches can efficiently match the shape of receptive fields in the visual cortex and optimally show the enhanced center-surround inhibition effects on magnocellular LGN. The choice of the Gabor patch sizes for center-surround inhibition effects is in reference to the work by Murphy and Sillito, 1987.

**Figure 1:**
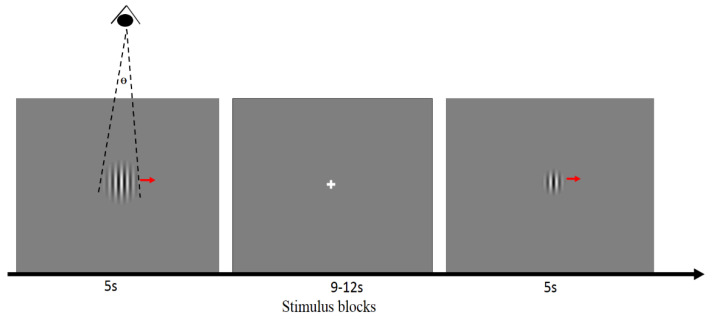
**An illustration of the experimental design**. The visual stimuli consisted of six varying sizes of rightward drifting Gabor patches (θ = 0.25°, 0.5°, 0.75°, 1°, 2°, 3°) and a white cross fixation. Each Gabor patch was displayed for 5 s, and the intervals were randomized to 9–12 s. The supplementary video file accompanying this report shows the stimulus as seen by the subjects.

The stimuli were generated on a Windows computer with MATLAB (The MathWorks Inc., Natick, MA, USA). Then, using E-prime (Psychology Software tools, Inc., https://www.pstnet.com/eprime.cfm accessed 20 January 2022) software, the stimuli were embedded into an event-related fMRI paradigm and displayed through an MR-compatible Avotec LCD projection system. The projector was located at the rear of the scanner room, and it projected the images onto a translucent screen attached to the inside of the scanner bore. The subjects viewed the projected images via a mirror mounted over the subjects’ eyes, with a total viewing distance of 113 to 115 cm. Since the screen position inside the scanner was fixed, the possible variability was the distance from the mirror to the subjects’ eyes. We measured the distance from the mirror to the center between eyes for each subject, and the estimated mean total viewing distance across all subjects was approximately 114 cm. The up–down range (height) of the screen subtended 10.5° of the visual angle, and the screen width subtended 13° of the visual angle. The stimuli consisted of six different sizes of rightward drifting gratings and a white cross fixation. Six varying sizes of moving sine wave gratings (Gabor patch) [31] over the receptive field were used to localize magnocellular LGN cells and evaluate the feedback effects on magnocellular LGN cells. The drifting Gabor patch stimuli were 50% luminance contrast, vertical black and white sinusoidal gratings with a spatial frequency of 1 cpd (cycle per degree). To maintain comparability, a 60 Hz display refresh rate, a 25 Hz frame rate, a constant stimulus horizontal velocity of 2.1° s−1 and constant screen brightness were used for all subjects. The outer borders of the patches faded into gray to avoid sharp edge effects. In order to obtain center-surround interactions, we had six varying sizes of patches corresponding to 0.25°, 0.5°, 0.75°, 1°, 2°, and 3° of visual angle (Figure 1). A centered white fixation cross was used between the patch stimuli, and it subtended 0.1° of the visual angle. The background was a gray screen. For each run, the display sequence of the six patches was randomized, and each patch size was repeated 6 times and shown for 5 s. The interval time for fixation between patches was randomized between 9 and 12 s (Figure 1). A video of the stimulus is included as a Supplementary File. In summary, for each subject, there were two runs, and 36 stimuli were shown in each run, lasting around 10 min. 

### 2.3. MRI Data Acquisition

High-resolution whole-brain anatomical images were acquired on a 7T Siemens MAGNETOM scanner with a 32 channel head coil (Nova Medical). The whole-brain high resolution three-dimensional (3D) MPRAGE sequence used the following parameters: 256 slices, voxel size: 0.6 mm × 0.6 mm × 0.6 mm, TR/TE: 2200/2.8 ms, 7° flip angle, base/phase resolution: 384/100%, in-plane phase-encode acceleration factor (iPAT) GRAPPA acceleration factor of 2, FOV read/ phase: 240 mm/100%, bandwidth: 270Hz/Px, ascending acquisition. 

Two separate high-resolution BOLD runs were obtained with a T2* weighted single-shot multiband gradient-echo echo planar imaging (EPI) sequence with the following parameters: 45 slices acquired parallel to the AC-PC line, voxel size: 0.7 mm × 0.7 mm × 1.5 mm, TR/TE: 1500/31 ms, 70° flip angle, FOV read 220 mm, base/phase resolution of 260/100%, the anterior-to-posterior phase encoding direction, in-plane phase-encode (iPAT) GRAPPA acceleration factor of 3, multiband (MB) slice acceleration factor of 3, partial Fourier of 6/8, echo spacing of 1 ms, interleaved acquisition, 366 measurements. 

Before entering the scanner, the subjects were instructed to pay attention to the drifting of the Gabor patch. Inside the scanner, subjects lay in the supine position with foam padding around the head to reduce head motion. To reduce fatigue effects, we gave the subjects 5 min rest with eyes closed inside the scanner between two fMRI runs. No data were acquired during this resting period.

### 2.4. Laminar Surface Reconstruction Using High-Resolution Anatomical MRI

**Figure 2:**
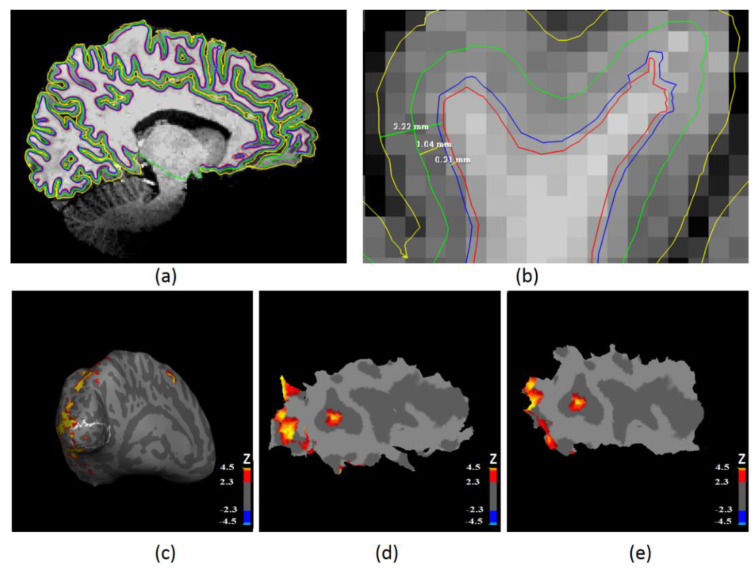
**Illustration of surface-based laminar analysis.** (**a**) Four laminar profiles overlaid on the original volume: white matter surface (red contour), layer VI surface (blue contour), layer IV surface (green contour), and pial surface (yellow contour). (**b**) A zoomed version of (**a**) illustrating the layers and their relative distances: the distance between the white matter surface to other laminar surfaces was as follows—0.21 mm (from white matter to layer VI, 10% of the thickness), 1.04 mm (from white matter to layer IV, 50% of the thickness), and 2.22 mm (from white matter to pial surface, 100% of the thickness). (**c**) Significant activation (Z > 2.3 and a FDR-corrected cluster significance threshold of *p* < 0.05) overlaid on inflated cortical surface. The white line shows the contour for the left primary visual cortex. (**d**) A flat patch consisting of significantly activated clusters (Z > 2.3, threshold at FDR corrected *p* < 0.05) on layer VI within left primary visual cortex. (**e**) A flat patch consisting of significantly activated clusters (Z > 2.3, threshold at FDR corrected *p* < 0.05) on layer IV within the left primary visual cortex.

To extract laminar functional MRI data, we need to first obtain cortical laminar profiles from anatomical MRI since it could provide better brain structural information. A very popular way to reconstruct cortical surfaces is using FreeSurfer’s (http://freesurfer.net/, accessed on 21 September 2022) automatic reconstruction pipeline [55]. However, this pipeline conforms the data to an isotropic resolution of 1 mm^3^. Because our MRI data had an isotropic resolution of 0.6 mm^3^, we applied Lüsebrink’s method [56] to process our 0.6 mm^3^ isotropic resolution data. This method avoids downsampling high-resolution MRI data (<1 mm^3^) through software modification of FreeSurfer’s standard processing pipeline. We then reconstructed the white matter and pial surfaces based on the original MRI resolution of 0.6 mm^3^. Laminar profiles were then extracted within the cortical gray matter surface. They were constructed at a fixed relative distance between the white and pial surfaces, which was determined from cortical thickness [47]. Two intermediate laminar surfaces were located at 10% and 50% of cortical thickness away from the white matter surface (Figure 2a,b), corresponding to layer VI and layer IV separately. The primary visual cortex was automatically identified with the FreeSurfer’s high-resolution data analysis pipeline [57]. The location of the primary visual cortex was confirmed by the cortical folds in a surface coordinate system. 

### 2.5. Functional MRI Analysis

The pre-processing steps for fMRI data followed standard procedures routinely employed for task fMRI data. All functional volumes from each run were aligned to the first volume to correct head motion using FSL software’s MCFLIRT functionality [58,59]. The Brain Extraction Tool (BET) was also used to remove non-brain tissues from functional images [60]. Then, the time series from each voxel was detrended to remove low-frequency noise and slow drift using high-pass temporal filtering (Gaussian-weighted least-squares straight line fitting, with sigma = 45 s). 

The statistical analysis to detect activated brain regions was carried out using FILM (FMRIB’s Improved Linear Model) functionality with local autocorrelation correction in FSL software [61]. The general linear modeling (GLM) method was used to estimate the response of each voxel to six different visual stimuli separately with a double-gamma HRF assumption for each single session. Z statistic images were thresholded using clusters determined by Z > 2.3 and a (corrected) cluster significance threshold of *p* < 0.05 (FDR corrected). The single-session first-level Z statistic maps were in each individual subject’s coordinate space. 

Since there were two separate task runs for each subject, we performed between-session higher-level analysis to estimate each subject’s mean response. The estimated responses of each voxel in each subject’s brain to the six different sizes of drifting Gabor patches corresponded to six distinct beta activation images (parameter estimate or PE images), respectively. The between-session mean PE maps for each subject are in standard MNI space. These between-session PE maps would only be used later to localize magnocellular neurons in LGN. Once the magnocellular LGN were localized, they were transformed to individual space, and all further analysis was carried out in individual subject space. 

### 2.6. Laminar Functional Data Analysis in the Primary Visual Cortex

To enable time series extraction or statistical analysis from these intermediate surfaces, it is essential to align the EPI volume to these surfaces. Here, we employed a boundary-based registration (BBR) method [62] in order to achieve this. It identified the boundary interface between the gray matter and white matter from EPI volumes and then calculated a 12 degrees of freedom affine transformation, which registered the boundary interface in EPI to the corresponding white matter surface, which was reconstructed from high-resolution anatomical MRI data. Careful manual inspections were carried out to check the accuracy of the registration and were manually edited when necessary. 

The single-session thresholded Z statistic images obtained from all six conditions were then transformed onto two intermediate reconstructed surfaces (they were located at 10% and 50% of the cortical thickness away from the white matter surface, corresponding to layers VI and IV, respectively) separately with the transformation matrix calculated above. Two corresponding Z statistic surfaces were then masked with the primary visual cortex region (Figure 2c), which was obtained from FreeSurfer [57]. 

For each session of each subject, two patches of cortical surface containing significantly activated clusters within the left or right primary visual cortex were identified and extracted from each intermediate surface, and then, they were flattened using a near-isometric flattening algorithm (Figure 2d,e) [63]. Then, we were able to extract and analyze time series from each flat patch ROI on each intermediate surface within the primary visual cortex. In summary, we extracted time series from activated regions of the left and right primary visual cortex separately for layer IV and layer VI. This analysis was performed in native subject space. 

### 2.7. LGN ROI Definition and Analysis

**Figure 3:**
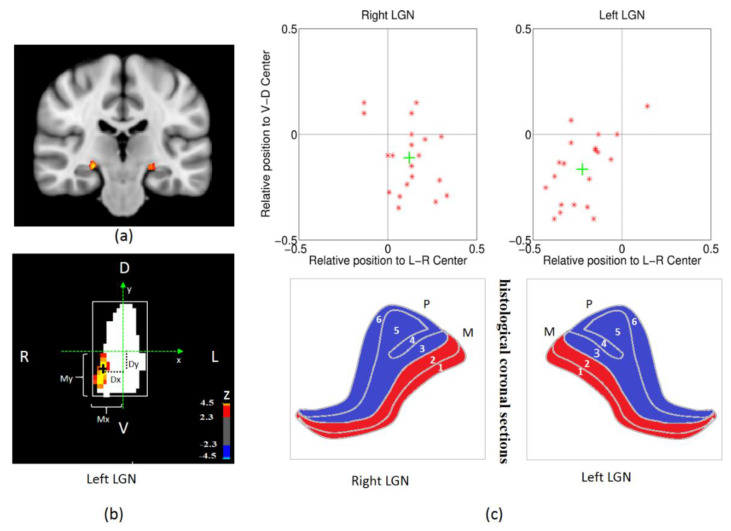
LGN definition and spatial analysis of top 20% activated voxels in LGN. (**a**) LGN mask from Juelich Histological Atlas (thresholded at 60%) overlaid onto MNI brain template. (**b**) The top 20% of activated voxels obtained with the 1° visual angle stimulus overlaid on the left LGN mask (the white region in (**b**) corresponds to the LGN mask shown in (**a**)) for one subject. The relative position of activated voxels is calculated as *Dx/Mx* and *Dy/My* where these quantities are depicted in (**b**). (**c**) The top panel plots the relative position of left and right magnocellular LGN identified in (**b**) with respect to the center for 20 subjects (red star) and the associated group average (green cross); the bottom panel shows the histological coronal sections of human LGN, the red layers represent magnocellular LGN and blue parts represent parvocellular LGN (referred and modified from [3]).

To investigate corticogeniculate feedback, localizing the LGN region of interest (ROI) is very important. This is not trivial, since LGN is a very small subcortical region. We first defined an LGN mask based on the Juelich Histological Atlas in MNI152_T1_1mm_brain coordinate space, thresholded at 60% [64]. As in previous studies [37], we defined the top 20% of activated voxels with the 1° visual angle stimulus condition to be our left or right magnocellular LGN ROI for each subject separately (Figure 3a,b). This specific stimulus condition was chosen because previous invasive studies in animals have shown that the response for 1° is expected to be highest compared with stimuli subtending other angles, as it corresponds to the size of the central receptive field for the magnocellular LGN neurons [16,17,19,24,41,65,66,67]. To assess the reliability of magnocellular LGN ROIs defined this way, the center coordinate of the left and right magnocellular LGN ROI for each subject was calculated in each spatial dimension (left–right, anterior–posterior, ventral–dorsal). These centers were in MNI152_T1_1mm_brain coordinate space. The relative spatial center (defined as *D_x_/M_x_* and *D_y_/M_y_*; please refer to Figure 2b for a visual illustration of what these quantities mean) was then calculated and plotted as a proportion of the extent away from the center of the LGN mask for left and right brain separately (Figure 3c). In principle, the magnocellular LGN center is expected to be close to the ventral direction. If the spatial center is more ventral, we could say the LGN ROI is more likely to be magnocellular LGN. 

After the definition of our magnocellular LGN ROIs for each subject, we converted the activation beta (PE) values to percentage change in order to help interpret the results and then extracted the mean percentage changes corresponding to each visual stimulus condition (0.25°, 0.5°, 0.75°, 1°, 2° and 3°) from the defined magnocellular LGN ROIs for each subject. Finally, the mean time series from magnocellular LGN ROIs were extracted. 

### 2.8. Dynamic Granger Causality Analysis

Granger causality is a technique used to quantify directional influences between brain regions [68,69,70]. The underlying principle is that the directional causal influence from region A to region B can be inferred if past values of region A help predict the present and future values of region B [71]. One form of Granger causality uses multivariate autoregressive models (MVAR) to characterize the predictive relationship between time series [72,73,74,75,76,77,78,79]. In the “classical” form of Granger causality used in these previous studies, the model coefficients are independent of time, and hence, the model is “static”. Consequently, it does not capture nonstationarities in fMRI time series [80] or the spatiotemporal dynamics of different layers under spontaneous cortical activity and evoked activity [81]. In addition, with static models, it is difficult to delineate the contributions of spontaneous and evoked activity toward the estimated connectivity value. Therefore, we employed a variation of the MVAR model wherein the model coefficients are a function of time [82,83,84]. This allowed us to calculate dynamic Granger causality (DGC) [85,86]. Specifically, in DGC, coefficients A(p) of the MVAR model are allowed to vary over time [87,88,89], therefore, giving A(p,t) in the model as:

Y(t)=A(0,t)Y(t)+A(1,t)Y(t−1)+…+A(p,t)Y(t−p)+E(t)

where *Y(t)* is a matrix containing the *k* different time series, *p* is the model order, *t* is time and *E(t)* is the model error. The diagonal elements of A(0,t) are set to zero, while the non-diagonal elements model the instantaneous influences between time series to compensate for zero-lag cross correlation effects [90]. The dynamic coefficients are estimated in a Kalman filter framework using variable parameter regression [91,92]. The DGC is then estimated as [53,54]:

$$DGC_{ij}\left(t\right)=\overset{p}{\underset{n=1}{\sum }}a_{ij}^{\prime }\left(n,t\right)$$

where $DGC_{ij}\left(t\right)$ is the effective connectivity value from ROI *i* to ROI *j* at a given time point *t*. $a_{ij}^{\prime }$ are the elements of matrix A. 

Recent studies have shown that the variability of hemodynamic response across brain regions can be a confounding factor for Granger causality analysis using raw fMRI time series [90,93]. Therefore, we performed blind hemodynamic deconvolution of raw BOLD time series to obtain latent neural signals and used them in DGC estimation. This approach has been employed and validated in multiple previous studies [93,94,95,96,97,98]. We employed a recently validated framework based on the Cubature Kalman filter and smoother to invert a nonlinear hemodynamic model [50]. Even though this model is highly parameterized, recent research indicates that it does not overfit the data [99]. 

**Figure 4:**
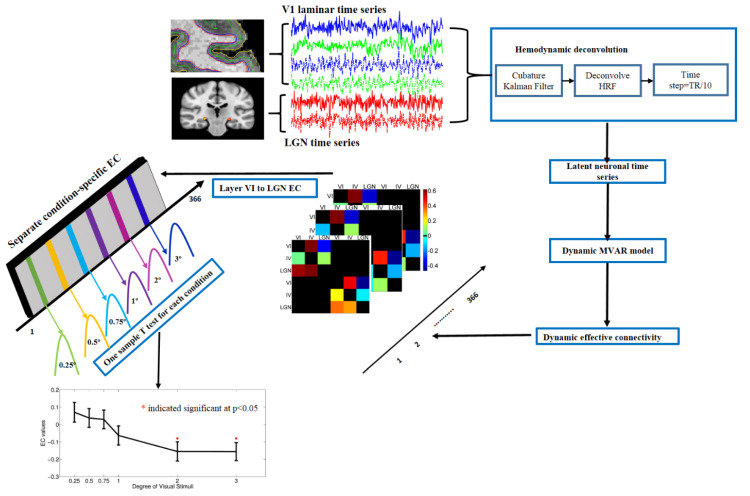
An illustration of the data processing pipeline. First, we performed surface-based laminar analysis including laminar surface reconstruction and the registration of functional MRI data to the laminar surfaces. Second, we extracted mean time series from activated vertices (voxels in the volume become vertices on a surface) within each laminar surface and the magnocellular LGN ROIs. Third, blind deconvolution was performed to obtain latent neuronal time series. Fourth, we utilized the dynamic MVAR model to obtain dynamic effective connectivity (one directional connectivity matrix for each time point; the connectivity direction is from row to column). Fifth, separation of condition-specific effective connectivity (18 × 40 EC values for each condition) for each path. An example is shown for the path from layer VI to LGN. Finally, we performed one sample T-test to determine paths whose strengths significantly differed from zero (red * indicates significant at FDR corrected *p* < 0.05).

In this study, we had six ROIs including layer IV and layer VI of the bilateral primary visual cortex and magnocellular LGN ROI for the left and right brain separately. After the extraction of average time series from these 6 ROIs, they were deconvolved as mentioned above and input into the DGC model. We obtained a 6 × 6 × 366 connectivity matrix (366 time points) for every run of every subject by employing DGC. Finally, DGC values corresponding to specific conditions of interest were populated into different samples; e.g., we grouped all DGC values for 18 time points corresponding to the 1° Gabor patch condition (6 × 6 × 18 matrix) for each run. According to our data, 40 runs were included into our analysis (2 runs each for 20 subjects). Therefore, we obtained a 6 × 6 × 18 × 40 connectivity matrix for each experimental condition. This matrix was then resized to 6 × 6 × 720 for each condition by merging connectivity values obtained across all runs and subjects for each path and condition. Statistical tests were carried out using the third dimension of this matrix as the sample in order to compare different conditions for our paths of interest. Figure 4 schematically illustrates the pipeline of the data processing.

## 3. Results

### 3.1. The Validity of the Spatial Localization of Magnocellular Neurons in LGN

We defined the top 20% of activated voxels with a 1° visual angle stimulus condition (Figure 1) to be our left or right magnocellular LGN ROI (region of interest) for each subject separately. This specific stimulus condition was chosen because previous invasive studies in animals have shown that the response for 1° is expected to be highest compared with stimuli subtending other angles, as it corresponds to the size of the central receptive field for the magnocellular LGN neurons [16,17,19,24,41,65,66,67]. We then located the spatial center of the magnocellular LGN ROIs in each spatial dimension (left–right, anterior–posterior, ventral–dorsal). These centers were in MNI152 coordinate space. The relative spatial center (defined as *D_x_/M_x_* and *D_y_/M_y_*; please refer to Figure 3b for a visual illustration of what these quantities mean) was then calculated and plotted as a proportion of the extent away from the center of the LGN mask for left and right LGN separately (Figure 3c). 

As we can see from Figure 3c, the group-averaged spatial center of the activated magnocellular LGN cluster (green cross) is prone to be more ventral for both the right and left side, which is consistent with histological studies. To some extent, this means those activated neurons within LGN are more likely to be magnocellular neurons. Therefore, the functional localization of magnocellular neurons in LGN with MRI is a feasible method, since it matches histological findings obtained from the human LGN [3]. 

### 3.2. The Enhanced Center-Surround Inhibition Effect on LGN

**Figure 5:**
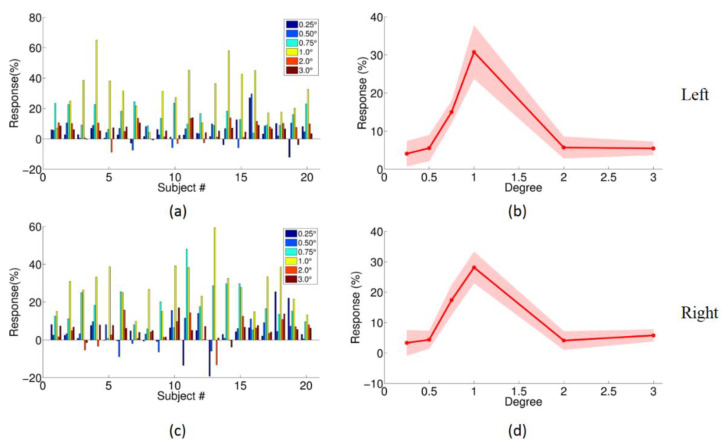
The BOLD response in magnocellular LGN for Gabor patch stimuli. (**a**,**b**) correspond to left magnocellular LGN, and (**c**,**d**) correspond to right magnocellular LGN. (**a**,**c**) the responses of each subject for each visual stimuli (0.25° dark blue, 0.5° blue, 0.75° cyan, 1° yellow, 2° red, 3° dark red), x axis is subject number, and y axis is the response (the percentage of change); (**b**,**d**) the plot of the mean response over all subjects vs. stimulus degree (red line), 95% confidence interval (red shade).

We extracted the mean percentage signal changes corresponding to each visual stimulus condition (0.25°, 0.5°, 0.75°, 1°, 2° and 3°) from the defined magnocellular LGN ROIs for each subject. The BOLD response in magnocellular LGN increased with the size of rightward moving Gabor patches until the stimulus attained a 1° angle and then decreased with subsequent increase in the size of the Gabor patch. Here, it is noteworthy that a 1° stimulus corresponds to the size of the central receptive field for the magnocellular LGN neurons [16,17,67,100,101], and hence, increasing the size of the patch beyond that meant that it breached in the surrounding receptive field. Consequently, if center-surround inhibition was in place, then increasing the size of the Gabor patch beyond 1 degree should decrease the BOLD response due to surround inhibition. As shown in Figure 5b,d, this is exactly what we found. Statistical comparison of the BOLD response elicited from each condition is shown in Table 1 (we only show comparisons for corrected *p* < 0.05, FDR corrected). The results showed that the responses within magnocellular LGN under both 0.75° and 1° visual stimuli were significantly greater than the responses under other conditions (Table 1). The maximum center-surround suppression (defined as the difference in BOLD response between 1°, and 2° or 3°, whichever is higher, as in previous studies [16,17,102]) at the individual subject level reached up to 59.67% for left magnocellular LGN (subject 4, shown in Figure 5a and Supplementary Figure S1) and 78.42% for right magnocellular LGN (subject 13, Figure 5c and Supplementary Figure S1). From Supplementary Figure S1, more than 20% center-surround inhibition (the bigger difference between 1° and 2° or 3° condition) in both left and right magnocellular LGN was found from 13 out of 20 subjects. Previous invasive studies in animals found center-surround inhibition of approximately 20% in magnocellular neurons within the LGN in the absence of negative feedback from the primary visual cortex [19]. Therefore, 20% inhibition represents a null benchmark against which we could compare the percentage inhibition obtained by us. Though individual differences exist, by and large at the group level, the enhanced center-surround inhibition effect on magnocellular neurons within human LGN is salient and detectable using noninvasive methods such as functional MRI. To our knowledge, this is the first study to detect the enhanced center-surround inhibition in human LGN noninvasively using fMRI. 

### 3.3. Center-Surround Inhibition Effects in Different Layers of Primary Visual Cortex

**Figure 6:**
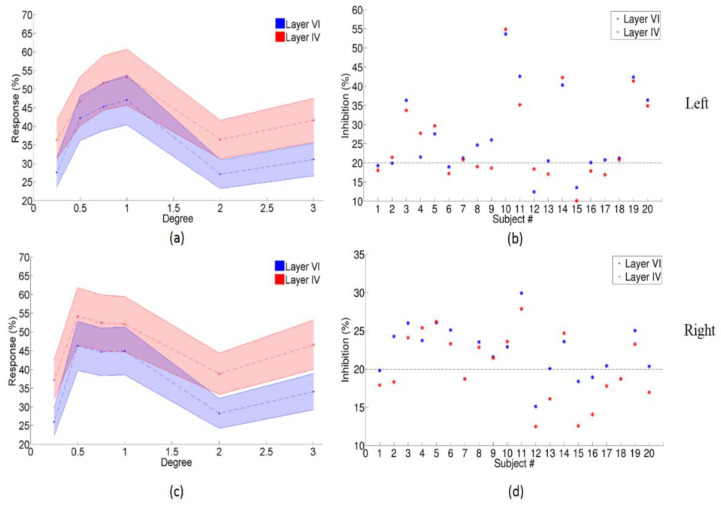
Center-surround inhibition in different layers of the primary visual cortex. Panel (**a**) shows the results for the left primary visual cortex and panel (**c**) shows the results for the right primary visual cortex. In both these panels, the mean BOLD response across all subjects (dash line) is plotted on the y-axis and the stimulus degree is plotted on the x-axis. The 95% confidence interval is shown as the shadow around the dash line. Red represents layer IV, and blue represents layer VI. Panels (**b**,**d**) show the center-surround inhibition (the larger of the difference in responses (1°–2°) or (1°–3°)) in each subject for the left and right primary visual cortex, respectively. Here, the red * represents layer IV, and blue * represents layer VI.

Two flattened patches of cortical surface containing significantly activated clusters within the left or right primary visual cortex were identified and extracted from each intermediate surface (Figure 2). Within the defined ROIs of the primary visual cortex, the mean percentage signal changes corresponding to each visual stimulus condition for each subject were determined. We found center-surround inhibition in primary visual cortical neurons as shown in Figure 6a,c and Table.2. The maximum center-surround inhibition (the larger of the difference in responses (1°–2°) or (1°–3°)) reached up to 54.86% for layer IV and 53.62 % for layer VI of the left primary visual cortex (Figure 6b, subject 10), 29.92% for layer IV, and 27.84% for layer VI of the right primary visual cortex (Figure 6d, subject 11). More than 20% center-surround inhibition was found in 14 subjects within the left primary visual cortex (Figure 6b) and in 10 subjects within the right primary visual cortex (Figure 6d). A comparison of the BOLD response due to different stimulus conditions for layer VI (shown in Table 2a and Table 3a for left and right primary visual cortex, respectively) and layer IV (shown in Table 2b and Table 3b for left and right primary visual cortex, respectively) within the primary visual cortex showed that the responses for 0.5°, 0.75°, and 1° conditions were significantly greater than the response for 0.25°, 2°, and 3° stimuli. Moreover, the fMRI response in layer IV was stronger than that in layer VI during the display of six different types of stimuli targeted at the magnocellular visual pathway (Table 2c and Table 3c). In addition, using a two-way ANOVA, we assessed the main effects of two different factors—visual stimuli and layers. We found both main effects to be significant (*p* < 0.05 FDR corrected), but the interaction between them was not (Table 4). 

### 3.4. Dynamic Effective Connectivity

We employed a surface-based laminar analysis pipeline to extract fMRI time series from the identified magnocellular LGN and primary visual cortex ROIs separately. With the raw fMRI time series, we performed hemodynamic deconvolution to recover the latent neuronal time series. Then, we fed the obtained latent neuronal time series into the dynamic MVAR (multivariate autoregressive) model to estimate the directional connectivity between magnocellular LGN and layers IV and VI of the primary visual cortex at each time point (i.e., dynamic effective connectivity). Subsequently, we separated the dynamic effective connectivity according to conditions (Figure 4) to obtain a sample distribution of directional connectivity for every condition, pathway and subject. As described in the Methods section, we obtained 720 (18 × 40, 18 time points for each run, 40 runs corresponding to 2 runs in 20 subjects) different effective connectivity values for each pathway, under each condition. We specifically extracted all effective connectivity values for the corticogeniculate feedback pathway and the three feedforward pathways under six conditions only in subjects who showed center-surround inhibition in their BOLD response above 20%. 

The major goal of this study was to determine whether the effective connectivity determined from layer-resolved fMRI in humans is sensitive to the negative feedback from layer VI of the primary visual cortex to magnocellular LGN. This negative feedback has been previously indicated by invasive animal studies [16,17,65,67] and is known to cause an enhanced center-surround inhibition effect on magnocellular LGN (demonstrated in the previous section). Additionally, we also wanted to investigate the feedforward pathway from magnocellular LGN to layers IV and VI in the primary visual cortex as well as that from layer IV to layer VI within the primary visual cortex (shown in Figure 6). Therefore, we investigated whether these pathways can be inferred by employing the dynamic effective connectivity modeling of layer-resolved human fMRI data. 

**Figure 7:**
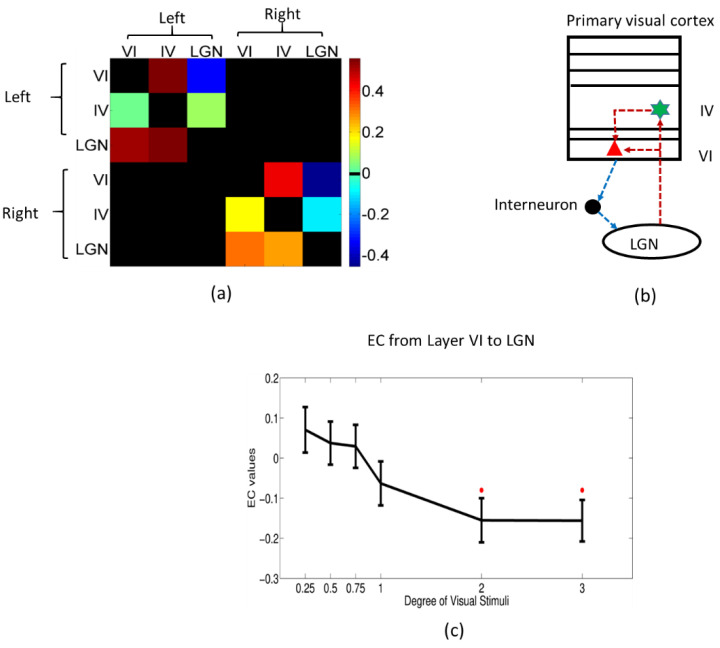
Dynamic effective connectivity results. (**a**) One example of the connectivity matrix at a given time point, the direction is from row to column, e.g., the left corticogeniculate feedback pathway from layer VI to LGN corresponds to the first row/third column, and the left feedforward pathway from layer VI to IV corresponds to the first row/second column. (**b**) An illustration of the neuronal circuits involving LGN and primary visual cortex: the black round shape represents an inhibitory interneuron, the red triangle is a neuron in layer VI of the primary visual cortex, and the green star is a neuron in layer IV. The blue dotted line represents the negative feedback pathway, and the red dotted lines are the feedforward pathways (LGN → IV, LGN → VI, and IV → VI). (**c**) Mean/standard deviations of effective connectivity values vs. stimulus degree for corticogeniculate feedback pathway from layer VI to LGN. This pathway is only significantly smaller than zero under 2° and 3° conditions. The red star indicates significance at FDR corrected *p* < 0.05.

First, the corticogeniculate feedback pathway from the primary visual cortex layer VI to magnocellular LGN was investigated under six different conditions. One-sample t-tests (stimulus > rest) were conducted for all six conditions; however, we found this feedback pathway to be significantly negative only for the 2° and 3° conditions Figure 7c). Under the 2° condition, mean connectivity was -0.155, t (719) = −3.2142, *p* = 0.0014; and under the 3° condition, mean connectivity values of −0.154, t (719) = −2.6536, *p* = 0.0083 were observed. This demonstrates that this negative feedback pathway is significantly enhanced when the receptive field of the visual stimuli exceeds its center (which happens when the Gabor path tends an angle of 2° or more) and corroborates the sharp drop in BOLD response observed in magnocellular LGN (Figure 5). 

**Figure 8:**
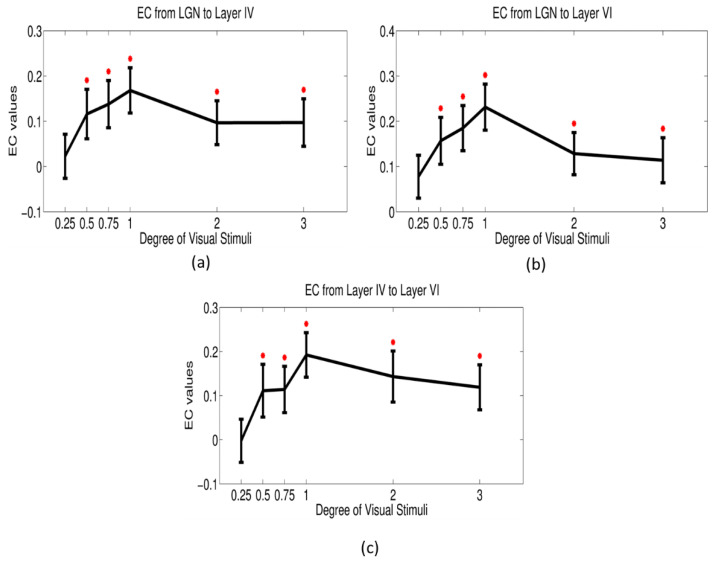
Mean/standard deviations of effective connectivity values vs. stimulus degree for feedforward pathway from LGN to layer IV in the primary visual cortex (**a**), LGN to layer VI (**b**), and layer IV to VI (**c**).

Subsequently, we performed a similar one-sample t-test for the feedforward pathways—from LGN to layers IV and VI in the primary visual cortex, and from layer IV to VI within the primary visual cortex (Figure 8). We found these positive feedforward pathways to be significantly greater than zero for all conditions except the 0.25° condition. This pattern mimicked that of the BOLD response within the magnocellular LGN and the primary visual cortex wherein the connectivity increased from 0.25° condition onwards, peaking at 1° condition and then decreasing. 

## 4. Discussion

The results presented in the previous section point to the following conclusions which we will discuss here: (1) functional localization of magnocellular neurons in LGN with high-resolution (<1 mm) fMRI has been demonstrated to be a feasible method; (2) the enhanced center-surround inhibition effect on magnocellular neurons within human LGN is salient and detectable using BOLD responses within the magnocellular LGN to moving Gabor patch stimuli of different sizes, being shown here for the first time in the literature; (3) with high-resolution, layer-specific fMRI at ultra-high fields, the effective connectivity determined from laminar fMRI is sensitive to the corticogeniculate negative feedback pathway from layer VI of the primary visual cortex to magnocellular LGN, and the feedforward positive pathways from magnocellular LGN to layers IV and VI of the primary visual cortex as well as from layer IV to layer VI within the primary visual cortex. To our knowledge, this is the first study to investigate whether fMRI-based effective connectivity can be used to investigate the directional information flow between cortical layers at the submillimeter spatial scale (and more specifically in the corticogeniculate pathway). 

Many previous studies have tried to use noninvasive methods such as fMRI to functionally localize small subcortical regions such as the LGN in the human brain [37,103,104,105,106,107,108,109]. However, only a few of those studies have been successful in identifying the magnocellular part of LGN [37,108,110]. These studies attempted to classify the magnocellular and parvocellular voxels in LGN based on the contrast sensitivity difference—magnocellular LGN response saturates under monochrome, high luminance contrast visual stimuli, and parvocellular LGN is more receptive to visual stimuli with high color contrast and low luminance contrast [10]. The voxel size used in these studies was around 2 mm3, which is pretty large compared to the size of the magnocellular and parvocellular parcels within LGN. In this study, we designed specific visual stimuli, which could maximally activate and help localize the magnocellular voxels in LGN with our whole-brain submillimeter fMRI data. We validated the location of magnocellular LGN using the expected spatial position obtained from previous histological findings [111]. 

Notably, this is the first study to detect the enhancement of the center-surround inhibition effect in magnocellular neurons within human LGN using fMRI. The maximum center-surround inhibition could reach up to 59.67% for left magnocellular LGN and 78.42% for right magnocellular LGN in individual subjects; however, the group mean center-surround suppression was only up to 26.52%. It did not reach up to 70% suppression observed in previous invasive animal studies [16,112], in which a single-unit recording was used to measure the inhibition from multiple neurons. In contrast, with ultra-high resolution fMRI, the inhibition is estimated from a small cluster, which may contain thousands of neurons. Therefore, the inability to accurately localize magnocellular neurons with fMRI (which is not surprising given that even the smallest voxel size achievable with fMRI is an order of magnitude larger than that needed to accurately localize magnocellular neurons) may have played a part in the magnitude of center-surround inhibition we observed at the group level. However, it is still very impressive that we were able to observe enhanced center-surround inhibition in human magnocellular LGN using fMRI. 

Previous invasive studies have found a dense network of feedforward and feedback projections between LGN and the primary visual cortex [1,2,3,4,5,6,7,20,21,22,23,24]. After neurons in LGN receive visual input from the retina, feedforward pathways would relay it to layers IV and VI of the primary visual cortex. The feedback pathway consists of corticogeniculate neurons in layer VI projecting onto the magnocellular neurons in the LGN via inhibitory interneurons. In this work, we employed a dynamic effective connectivity model to reveal the directional information flow for the feedforward and feedback pathways, with high-resolution, layer-specific fMRI data. We found a negative corticogeniculate feedback pathway from layer VI to magnocellular LGN (Figure 7) when the angle subtended by the Gabor patch stimulus was larger than the central visual field, thereby facilitating center-surround inhibition. It is noteworthy that negative effective connectivity from layer VI to magnocellular LGN implies that an increase in signal amplitude in layer VI predicts a decrease in signal amplitude in magnocellular LGN. This might explain the sharp drop in the BOLD response we observed in magnocellular LGN for larger Gabor patches (Figure 5). 

We also found positive feedforward pathways from magnocellular LGN to layers IV and VI of the primary visual cortex as well as from layer IV to layer VI within the primary visual cortex (Figure 8). It is interesting to note that those feedforward pathways mimicked the center-surround pattern of BOLD response observed in LGN. Therefore, we speculate that this might be a mechanistic explanation for how the response in one brain region is propagated to another, specifically from LGN to the primary visual cortex in our case. 

These findings indicate that layer VI of the primary visual cortex has a crucial role in controlling the gain of brain activity involved with vision. The tuning of the gain in the corticogeniculate control system is achieved by both feedback and feedforward pathways anchored by layer VI in the primary visual cortex. Similar conclusions about gain control by layer VI were reached by Olsen et al. by performing invasive electrical recordings in mice by using a technique that can selectively label neurons in layer VI ^24^. It is very encouraging that it is now possible to derive mechanistic insights about cortical layer-specific micro-circuits using fMRI which agree with the gold standard in neuroscience, viz. invasive single unit recordings. 

Our framework is domain-neutral; i.e., it can easily be extended to study layer-specific microcircuits in other brain systems. The cortical laminar separation was carried out using whole brain data for each individual. Further, the connectivity model was agnostic about the context in which it was applied and was not fine-tuned in any way to suit the network we were trying to characterize. The only part of the analysis pipeline that was not domain-neutral was the experimental and analysis paradigm used to localize magnocellular neurons in the LGN. Therefore, our framework could potentially be used to investigate the laminar connectional architecture anywhere in the human brain as long as an experimental paradigm is developed to localize the regions/layers of interest using high-resolution fMRI. For example, future studies could potentially investigate the laminar connections between Broca’s area to language-related thalamic nuclei. Broca’s area and language-related thalamic nuclei are connected in two parallel layer-specific pathways: one thalamic pathway targets the middle cortical layers in Broca’s area, and the other projects to cortical layer I. The feedback pathway from Broca’s area to thalamic nuclei originates from cortical layers V and VI. Neuroimaging studies could investigate these pathways from aphasic patients with damage to the thalamic nuclei [113]. The language function is unique to humans (like many other social and cognitive functions), and therefore, it is difficult to investigate such layer-specific functional microcircuits with invasive studies in animals. 

## 5. Limitations and Future Work

Laminar effective connectivity may provide novel insights into cortical microcircuits in the human brain connectome; however, a few limitations of this study need to be addressed in future laminar fMRI effective connectivity-related research. First, different methods to differentiate cortical lamina from MRI exist. In this study, we constructed laminar surfaces by a method called equidistant laminae, which keeps a relatively fixed distance to cortical boundaries [46,114,115]. Laminar surfaces can also be constructed along equipotentials, which are computed between the inner white matter surface and pial surface with the Laplace equation [48]. Moreover, a new model called the equal-volume model for identifying cortical laminae was proposed by Waehnert and colleagues [49]. In the future, studies must compare the three different models for how well the connectivity derived from layers constructed by them matches the underlying anatomical predictions. 

Second, the spatial point spread function (PSF) of the BOLD response at different layers presents poor laminar specificity, since the draining blood flows along the intracortical veins (ICV) (ICVs are perpendicular to the surface) into pial veins on the pial surface [116]. This shows that the lower layers always contribute a signal to the upper layers. The traditional method to resolve this issue is to interpolate the fMRI signal at certain cortical depths and then average the surface profiles [43,46,47]. The fact that we performed hemodynamic deconvolution before connectivity analysis also helps in removing some of the contributions of vasculature [98]. However, we need to come up with a better model to extract laminar signals to increase the spatial specificity of fMRI. Future studies may employ spin echo sequences to investigate whether they are better for laminar fMRI analysis since the PSF for spin-echo EPI is more localized than gradient-echo EPI [116]. While one may lose sensitivity by using spin echo, the tradeoff between the sensitivity lost by spin echo and the spatial precision gained by its sharper PSF must be investigated.

Third, despite the small size of LGN, the spatial organization of the magnocellular neurons in LGN in our study exhibited the expected distribution from previous histological studies; i.e., the spatial center was biased toward the ventral direction [111]. Previous studies have investigated the ability of fMRI to localize magnocellular regions within human LGN [37,108]; however, the spatial resolution of these fMRI studies was relatively coarse (>1 mm). In this study, we acquired fMRI data with 0.7 mm^2^ in-plane resolution and validated the reliability of magnocellular LGN distribution across 20 subjects. Still, there are some aspects that could be improved. Since our voxel size was not isotropic, partial volume effects could be one confounding factor; i.e., a single voxel could potentially contain both magnocellular neurons and parvocellular/koniocellular neurons. In such a scenario, the response from one single voxel could be a mixture of signals from different types of neurons. Future studies need to use higher spatial resolution to reduce partial volume effects at 7T. This could potentially be completed using approaches which restrict the field of view to include just the LGN and the primary visual cortex (more generally, just the structures of interest in the pathways being investigated). We did not use this approach because the FreeSurfer analysis pipeline for surface-based analysis requires whole brain coverage. However, it should be possible in principle to implement this pipeline using data with limited coverage and field of view. In addition, we defined the top 20% of activated voxels as containing magnocellular neurons in this study. While this choice was indeed conservative and motivated by previous studies, the proportion has been shown to vary across individuals [111,117]. Therefore, future studies could potentially use multiple alternative MR contrasts such as susceptibility, proton density and myelination in order to fine tune the functionally defined magnocellular LGN region. 

Fourth, effective connectivity analysis using dynamic Granger causality (DGC) suffers from the poor temporal resolution of fMRI. Although we performed hemodynamic deconvolution to recover the latent neuronal signals before applying Granger causality, it is still hard to infer neuronal latency from fMRI data, which is sampled coarsely in time. In future work, simultaneous EEG and fast laminar fMRI may be fused together to improve temporal precision of fMRI at the layer level. Recent studies have shown that it is possible to obtain EEG data simultaneously with fMRI sampled at 600 ms using the multiband EPI sequence [118]. With higher acceleration factors, one could potentially acquire fMRI with sampling periods less than half a second, and this will likely improve the performance of DGC [51] for inferring directional interactions within the human brain at the laminar level.

## 6. Conclusions

In conclusion, our results demonstrated that: (1) the functional localization of magnocellular cells in LGN with high-resolution MRI is a feasible method; (2) the “enhanced center surround inhibition” effect on magnocellular cells within human LGN is salient and detectable using noninvasive high resolution functional MRI at 7T; (3) the feedforward and corticogeniculate feedback functional pathways can be inferred using dynamic directional connectivity models of fMRI and could potentially explain the mechanism underlying center-surround inhibition in the human visual system; (4) our framework is domain-neutral and could potentially be employed to investigate the human brain’s connectome at the laminar level in other systems related to cognition, memory and language.

## Figures and Tables

**Table 1:** Paired *t*-test between the BOLD responses obtained from different conditions (condition A < condition B) for left (**a**) and right (**b**) magnocellular LGN, shown here only for comparisons for which FDR corrected *p* < 0.05. The results showed that the BOLD response within magnocellular LGN for 0.75° and 1° visual stimuli was significantly greater than the responses under other conditions.

**(a)**		
**Paired *t*-Test for Condition A < Condition B at Alpha = 0.05 in Left M Type LGN**		
**A**	**B**	***p* Value**
0.25°	0.75°	2.40 × 10^−3^
0.5°	0.75°	3.60 × 10^−3^
2°	0.75°	8.99 × 10^−5^
3°	0.75°	9.32 × 10^−4^
0.25°	1°	2.07 × 10^−8^
0.5°	1°	2.07 × 10^−8^
2°	1°	2.21 × 10^−8^
3°	1°	3.07 × 10^−8^
**(b)**		
**Paired *t*-Test for Condition A < Condition B at Alpha = 0.05 in Right M Type LGN**		
**A**	**B**	***p* Value**
0.25°	0.75°	1.10 × 10^−3^
0.5°	0.75°	6.37 × 10^−7^
2°	0.75°	1.00 × 10^−4^
3°	0.75°	5.50 × 10^−8^
0.25°	1°	4.06 × 10^−6^
0.5°	1°	7.17 × 10^−6^
2°	1°	2.10 × 10^−3^
3°	1°	1.90 × 10^−3^

**Table 2:** Paired *t*-test between the BOLD responses (FDR corrected *p* < 0.05) obtained from different conditions (condition A < condition B) for layer VI (**a**) and layer IV (**b**) within the **left** primary visual cortex. (**c**) Paired *t*-test of the BOLD responses obtained from layers IV and VI under each stimulus condition within the left primary visual cortex. *p*-values correspond to paired *t*-tests conducted to test the following contrast: layer IV > VI at FDR corrected *p* < 0.05.

**(a)**					
**Paired *t*-Test for Condition A < Condition B in Layer VI of Left Primary Visual Cortex**					
**A**		**B**		***p* Value**	
0.25°		0.5°		1.98 × 10^−7^	
0.25°		0.75°		2.07 × 10^−8^	
0.25°		1°		2.39 × 10^−8^	
2°		0.5°		3.67 × 10^−5^	
2°		0.75°		2.07 × 10^−4^	
2°		1°		2.09 × 10^−8^	
3°		0.5°		7.18 × 10^−6^	
3°		0.75°		2.55 × 10^−8^	
3°		1°		1.87 × 10^−7^	
**(b)**					
**Paired *t*-Test for Condition A < Condition B in Layer IV of Left Primary Visual Cortex**					
**A**		**B**		***p* Value**	
0.25°		0.5°		1.98 × 10^−7^	
0.25°		0.75°		2.07 × 10^−8^	
0.25°		1°		2.39 × 10^−8^	
2°		0.5°		3.67 × 10^−5^	
2°		0.75°		2.07 × 10^−4^	
2°		1°		2.09 × 10^−8^	
3°		0.5°		7.18 × 10^−6^	
3°		0.75°		2.55 × 10^−8^	
3°		1°		1.87 × 10^−7^	
**(c)**					
***p*-Value of Paired *t*-Test for Layer IV > VI under Six Conditions**					
**0.25°**	**0.5°**	**0.75°**	**1°**	**2°**	**3°**
4.71 × 10^−12^	7.45 × 10^−5^	3.53 × 10^−8^	2.39 × 10^−8^	5.54 × 10^−12^	2.69 × 10^−10^

**Table 3:** Paired *t*-test between the BOLD responses (FDR corrected *p* < 0.05) obtained from different conditions (condition A < condition B) for layer VI (**a**) and layer IV (**b**) within **right** primary visual cortex. (**c**) Paired *t*-test of the BOLD responses obtained from layers IV and VI under each stimulus condition, within the right primary visual cortex. *p*-values correspond to paired *t*-tests conducted to test the following contrast: layer IV > VI at FDR corrected *p* < 0.05.

**(a)**					
**Paired *t*-Test for Condition A < Condition B in Layer VI of Right Primary Visual Cortex**					
**A**		**B**		***p* Value**	
0.25°		0.5°		2.07 × 10^−8^	
0.25°		0.75°		2.06 × 10^−8^	
0.25°		1°		2.06 × 10^−8^	
2°		0.5°		2.06 × 10^−5^	
2°		0.75°		2.06 × 10^−4^	
2°		1°		2.05 × 10^−8^	
3°		0.5°		2.08 × 10^−6^	
3°		0.75°		2.07 × 10^−8^	
3°		1°		3.10 × 10^−7^	
**(b)**					
**Paired *t*-Test for Condition A < Condition B in Layer IV of Right Primary Visual Cortex**					
**A**		**B**		***p* Value**	
0.25°		0.5°		2.44 × 10^−8^	
0.25°		0.75°		2.10 × 10^−8^	
0.25°		1°		2.28 × 10^−8^	
2°		0.5°		2.13 × 10^−8^	
2°		0.75°		4.87 × 10^−7^	
2°		1°		2.07 × 10^−8^	
3°		0.5°		1.19 × 10^−4^	
3°		0.75°		1.65 × 10^−5^	
3°		1°		4.60 × 10^−3^	
**(c)**					
***p*-Value of Paired *t*-Test for Layer IV > VI under Six Conditions**					
**0.25°**	**0.5°**	**0.75°**	**1°**	**2°**	**3°**
1.50 × 10^−14^	8.06 × 10^−9^	1.49 × 10^−9^	9.75 × 10^−9^	1.24 × 10^−14^	5.84 × 10^−12^

**Table 4:** Two-way ANOVA F test results (*p*-value) for left and right primary visual cortex separately. The two main factors—visual stimuli and layers—were both significant, but the interaction between them was not.

	Left Primary Visual Cortex	Right Primary Visual Cortex
Visual Stimuli Factor	3.02 × 10^−14^	3.10 × 10^−14^
Layer Factor	2.07 × 10^−6^	9.97 × 10^−20^
Interaction	0.87	0.40

## Data Availability

All data used in this work is available upon reasonable request to the corresponding author.

## Supplementary Materials

The following supporting information can be downloaded at https://www.mdpi.com/article/10.3390/brainsci12101361/s1, Figure S1: The maximum center-surround inhibition of left (*) and right (◊) magnocellular LGN for each subject. More than 20% center-surround inhibition (the bigger difference between 1° and 2° or between 1°and 3° condition) in both left and right magnocellular LGN was found in 13 out of 20 subjects; Video S1: 2° of rightward moving Gabor patch used in this experiment.
