# Supplementary material for: Noninvasive Characterization of Functional Pathways in Layer-Specific Microcircuits of the Human Brain Using 7T fMRI
Source: Brain Sci. 2022 Oct 7;12(10):1361. doi: 10.3390/brainsci12101361 (PMC9599333; doi:10.3390/brainsci12101361)
Supplement: Supplementary file 1 [file brainsci-12-01361-s001.zip › Supplementary Figure S1.pdf]

SUPPLEMENTARY MATERIAL FOR REVIEW

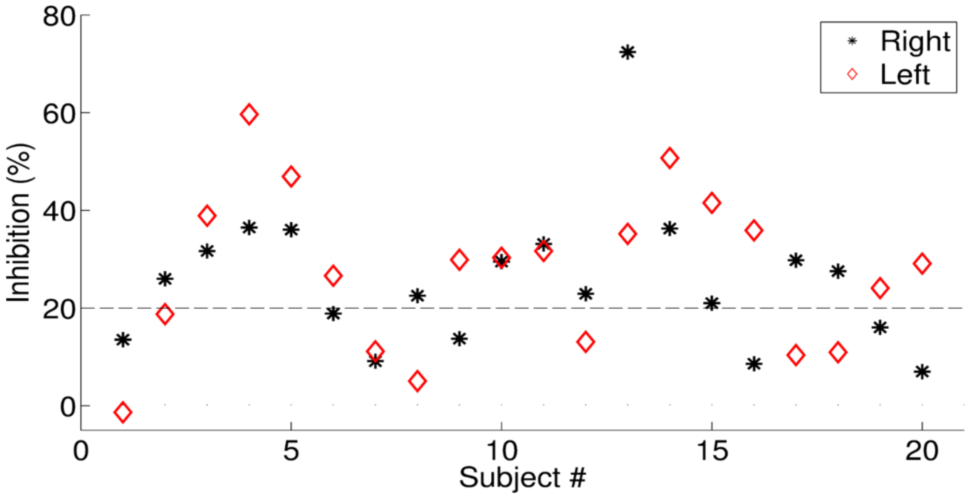

Supplementary Figure S1. *The maximum center-surround inhibition of left (*) and right (◊) magnocellular LGN for each subject. More than 20% center-surround inhibition (the bigger difference between 1° and 2° or between 1°and 3º condition) in both left and right magnocellular LGN was found in 13 out of 20 subjects.*
